# Supplementary material for: Ultra-wide-field, deep, adaptive two-photon microscopy for multi-scale neuronal imaging
Source: Light Sci Appl. 2026 Apr 13;15:198. doi: 10.1038/s41377-026-02252-2 (PMC13077073; doi:10.1038/s41377-026-02252-2)
Supplement: Supplementary file 1 — Supplemental Material [file 41377_2026_2252_MOESM1_ESM.pdf]

Supplementary Information for Ultra-wide-field, deep, adaptive two-photon microscopy for multi-scale neuronal imaging

A	X-axis		Y-axis		Z-axis	
Orientation	Mean	SD	Mean	SD	Mean	SD
Center	1.02	0.04	1.06	0.02	10.76	0.25

Ring B	X-axis		Y-axis		Z-axis	
Orientation	Mean	SD	Mean	SD	Mean	SD
0°	1.13	0.09	1.07	0.03	10.52	0.08
45°	1.17	0.06	1.08	0.02	10.84	0.15
90°	1.17	0.06	1.05	0.03	11.23	0.02
135°	1.149	0.08	1.04	0.02	10.79	0.17
180°	1.12	0.07	1.10	0.08	10.72	0.12
225°	1.18	0.11	1.06	0.03	10.61	0.06
270°	1.15	0.14	1.04	0.02	10.25	0.06
315°	1.16	0.11	1.05	0.02	11.17	0.13

Ring C	X-axis		Y-axis		Z-axis	
Orientation	Mean	SD	Mean	SD	Mean	SD
0°	1.11	0.10	1,09	0.01	10.65	0.15
45°	1.17	0.13	1.08	0.03	11.75	0.12
90°	1.28	0.05	1.03	0.03	10.77	0.11
135°	1.22	0.07	1.06	0.03	10.39	0.13
180°	1.19	0.11	1.09	0.77	10.13	0.19
225°	1.18	0.09	1.06	0.02	9.93	0.17
270°	1.23	0.10	1.05	0.03	10.79	0.17
315°	1.17	0.06	1.14	0.08	10.53	0.20

Ring D	X-axis		Y-axis		Z-axis	
Orientation	Mean	SD	Mean	SD	Mean	SD
0°	1.13	0.12	1.63	0.04	13.14	0.20
45°	1.32	0.02	1.28	0.05	13.99	0.26
90°	1.78	0.15	1.14	0.04	14.00	0.18
135°	1.39	0.06	1.26	0.05	13.50	0.21
180°	1.10	0.06	1.44	0.06	11.64	0.24
225°	1.21	0.11	1.21	0.04	12.52	0.22
270°	1.65	0.10	1.13	0.09	12.54	0.31
315°	1.29	0.09	1.27	0.02	13.16	0.21

Supplementary table 1 Summary of the resolution measurement across the whole FOV (all measurements are in μm)

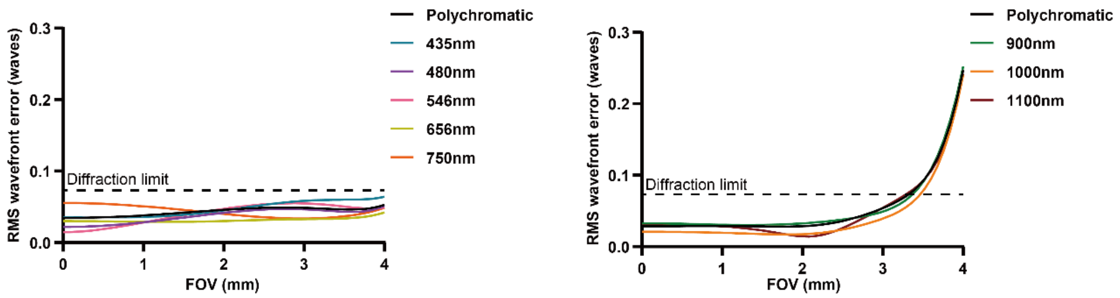

Supplementary Fig. 1 Simulated wavefront aberrations and diffraction-limited performance. Left, RMS wavefront error of the objective lens as a function of field of view (FOV) across the visible and short-NIR wavelengths (435–750 nm). The design maintains diffraction-limited performance (RMS error < 0.07 waves, indicated by the dashed horizontal line) across the entire 4 mm FOV. **Right**, RMS wavefront error of the integrated optical system (objective and tube lens) for near-infrared wavelengths (900–1100 nm). The system remains diffraction-limited within 85% of the full FOV (up to ~3.4 mm), ensuring high-fidelity signal collection for deep-tissue imaging. These results validate the achromatic and wide-field capabilities of the custom-designed optics for multi-modal neural recording.

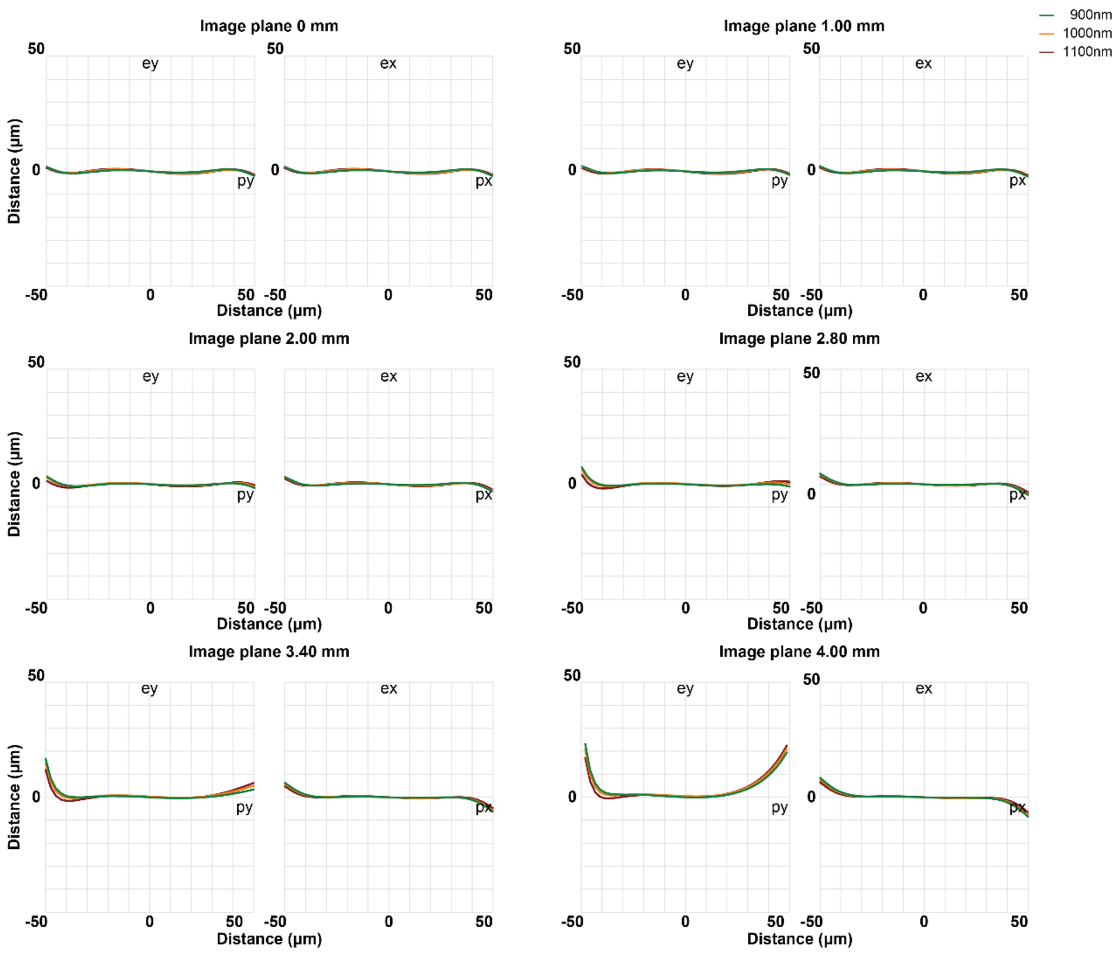

Supplementary Fig. 2 Optical aberration characterization. Simulated RMS wavefront error as a function of field of view (FOV) for the objective lens alone (435–750 nm) and the integrated system including the tube lens (900–1100 nm). The design achieves diffraction-limited performance across the entire 4 mm FOV for visible wavelengths and up to 85% FOV (~3.4 mm) for the NIR regime. Transverse ray aberration curves of the integrated system at representative field positions (0, 1.00, 2.00, 2.80, 3.40, and 4.00 mm) for NIR wavelengths (900, 1000, and 1100 nm). While the on-axis performance (0 mm) exhibits minimal residual spherical aberration, off-axis fields are primarily characterized by coma. Despite these aberrations, the system maintains high-fidelity imaging quality within the designated functional FOV.

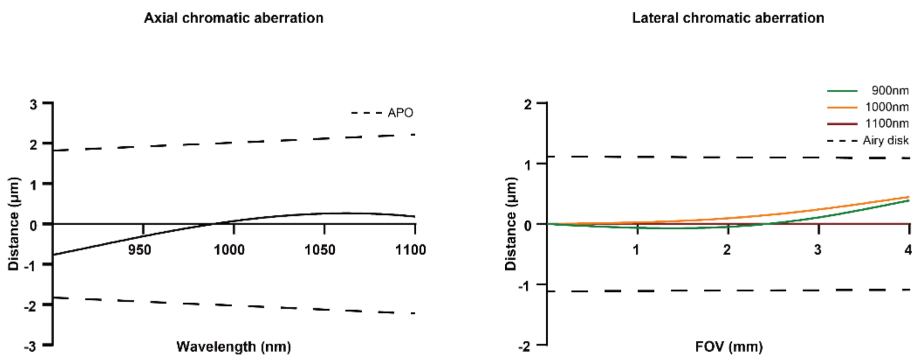

Supplementary Fig. 3 Chromatic aberration correction. Simulated spectral transmittance of the objective lens, showing >90% throughput from 435 to 1100 nm. **Left**, Axial chromatic aberration (longitudinal focal shift) of the objective-tube lens system from 900 to 1100 nm. The shift is calculated based on Zernike Z_4 = 0 and remains well within the APO tolerance limits (dashed lines). **Right**, Lateral chromatic aberration as a function of FOV for 900, 1000, and 1100 nm (referenced to 1100 nm). The lateral shifts across the entire FOV are contained within the Airy disk radius (dashed lines), ensuring spatial co-registration of multi-spectral NIR signals without computational alignment.

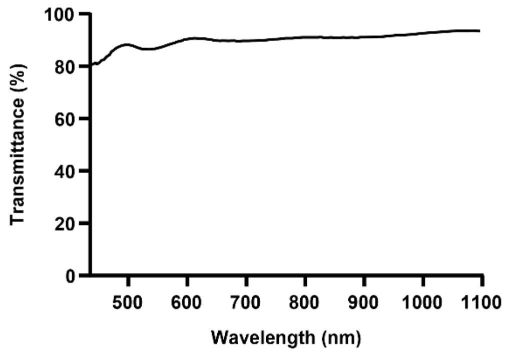

Supplementary Fig. 4 Simulated broadband spectral transmittance of the custom-designed objective. The simulated transmittance profile of the objective lens demonstrates high throughput (exceeding 90% the majority of the spectrum) over a broad spectral range from 435 nm to 1100 nm. This bandwidth covers the entire visible spectrum and extends into the NIR regime, ensuring efficient photon collection for multi-modal imaging and deep-tissue applications.

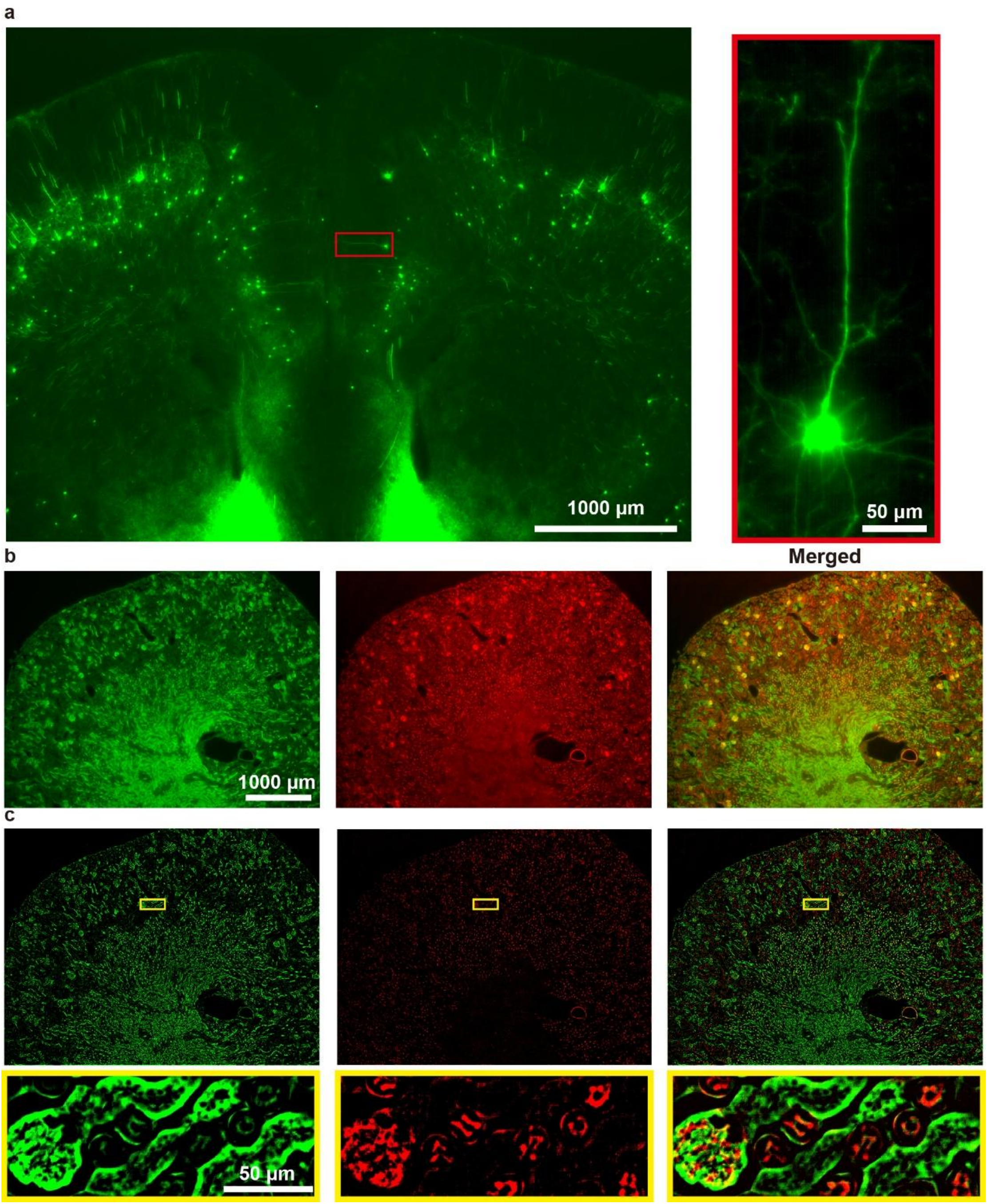

Supplementary Fig. 5 Wide-field fluorescence imaging of various tissue samples using ULTRA's objective. **a**, Wide-field fluorescence imaging of brain slice of Thy1-GFP transgenic mice (thickness 50 μm) pixel size 14192 x 10640, FOV 4807 μm x 3604 μm. **b**, Dual-color wide-field fluorescence imaging of mouse kidney (FluoCells™ Prepared Slide #3 (Cat. No. F24630)), elements of the glomeruli and convoluted tubules stained in green-fluorescent lectin and filamentous actin prevalently stained in red fluorescent phalloidin. **c**, Speckle illumination dual-color wide-field fluorescence imaging of same sample in **b** enhancing optical sectioning.

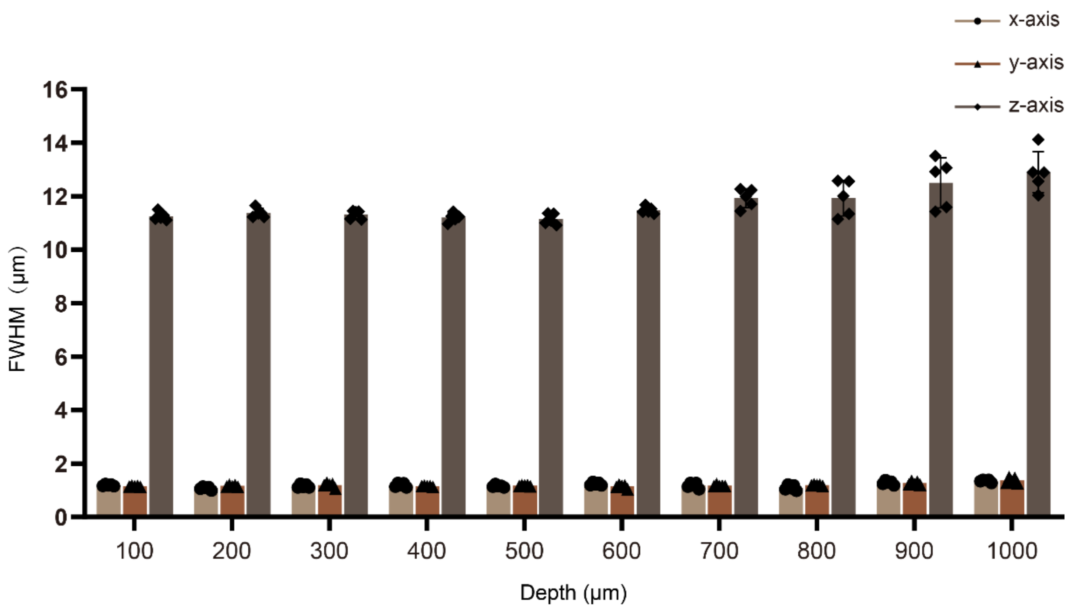

	X-aixs		Y-axis		Z-axis	
Depth	Mean	SD	Mean	SD	Mean	SD
0-100	1.19	0.03	1.15	0.02	11.25	0.16
100-200	1.08	0.06	1.16	0.04	11.37	0.17
200-300	1.16	0.06	1.20	0.08	11.32	0.16
300-400	1.20	0.07	1.15	0.02	11.20	0.17
400-500	1.16	0.05	1.18	0.02	11.14	0.20
500-600	1.24	0.06	1.15	0.07	11.48	0.13
600-700	1.18	0.10	1.17	0.03	11.93	0.35
700-800	1.10	0.10	1.20	0.02	11.93	0.67
800-900	1.29	0.07	1.27	0.06	12.50	0.93
900-1000	1.35	0.05	1.37	0.11	12.90	0.76

Supplementary Fig. 6 Resolution measurement in depth. 0.5-μm fluorescent microspheres embedded in a thick 0.75% agarose gel were imaged at the center of the FOV under the objectives and ten 100-μm z-stacks were acquired from 0 to 1000 μm deep in the sample (n=5). The FWHM of the Gaussian fits for measurements (mean ± SD) indicate both lateral and axial resolutions maintain good consistency as imaging depth increases, relative to the resolutions measured at the sample surface **(all measurements are in μm)**.

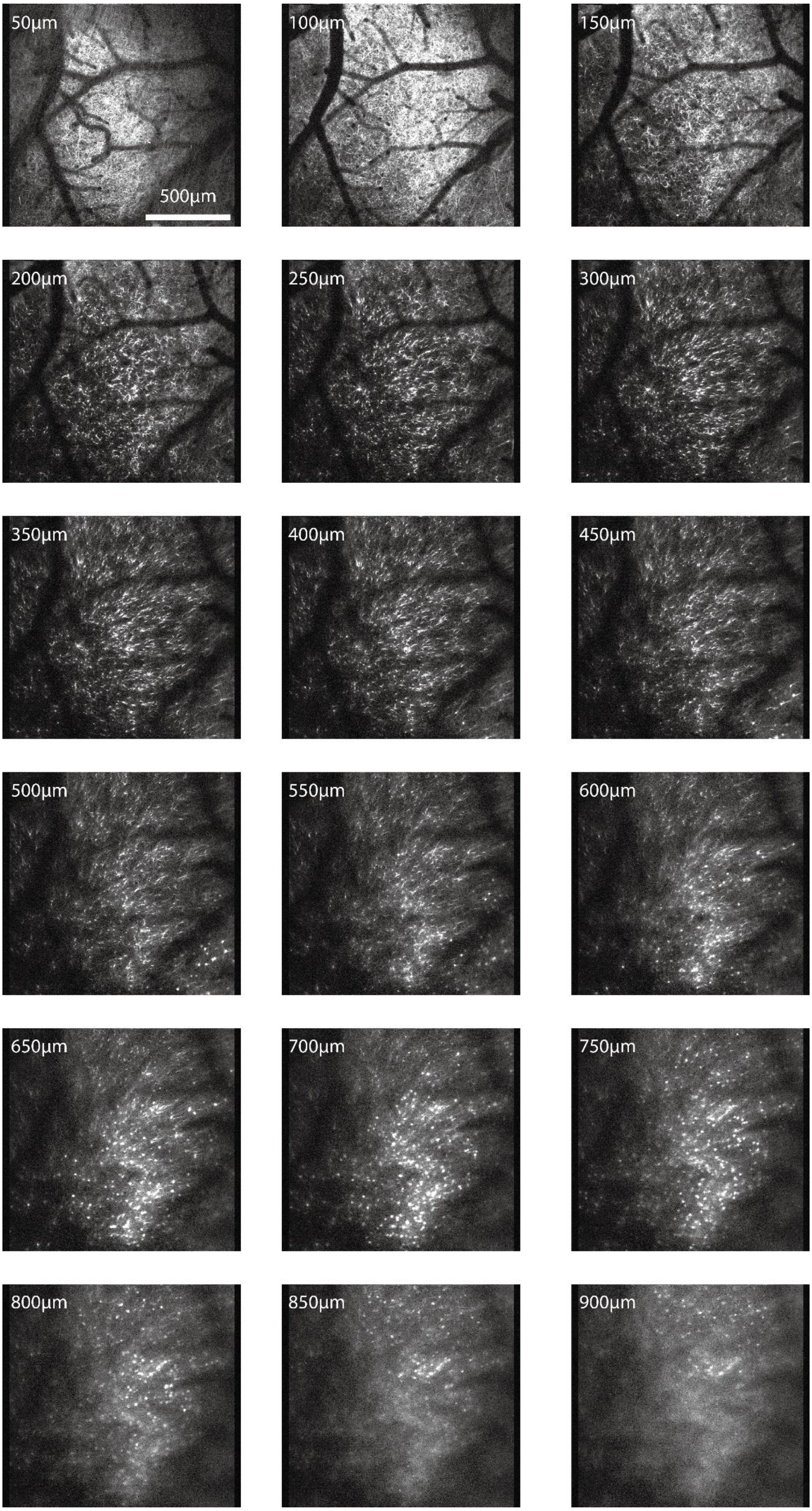
50µm
100µm
150µm
500µm
200µm
250µm
300µm
350µm
400µm
450µm
500µm
550µm
600µm
650µm
700µm
750µm
800µm
850µm
900µm

Supplementary Fig. 7 ULTRA z-stack imaging of dendrites ana somata up to 900 μm (50 μm per step) shown in Fig. 3b right.

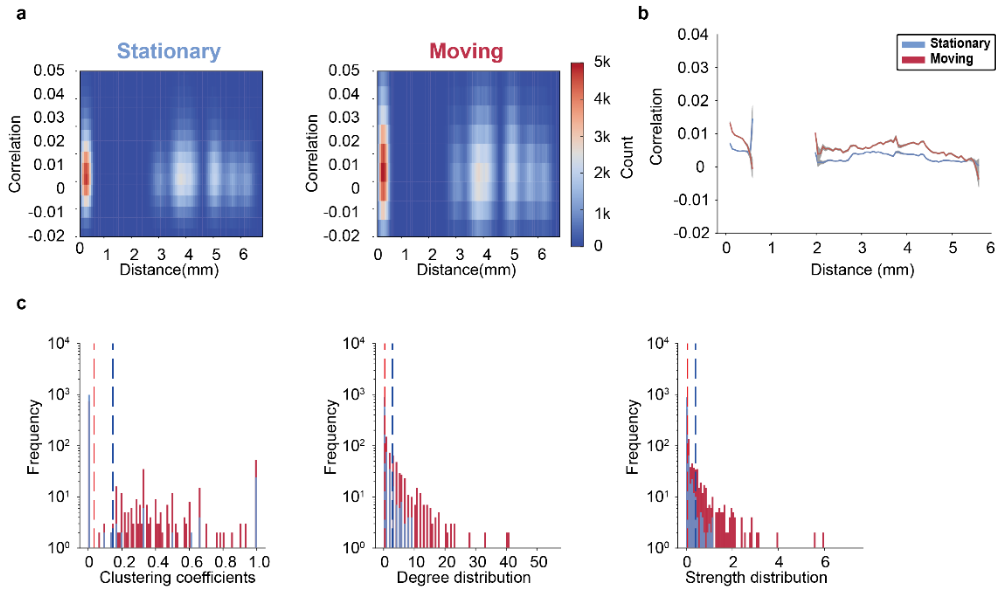

Supplementary Fig. 8 Functional connectivity assessments on neuronal ensembles across 4 independent regions. a, Distribution of correlation as a function of distance between neurons across different states. **b**, The trend of correlation as a function of distance between neurons across different states. **c**, Similar analysis to that in Fig. 5d with mean value (dashed line).

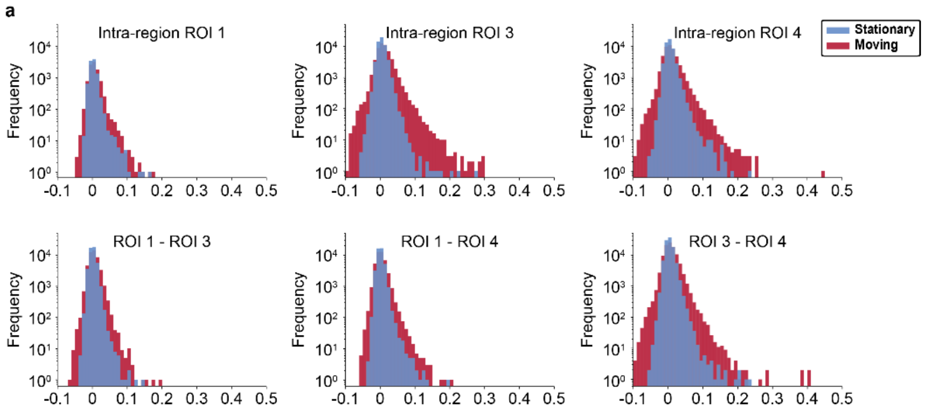

Supplementary Fig 9 Correlation distribution of intra-region and inter-region between states

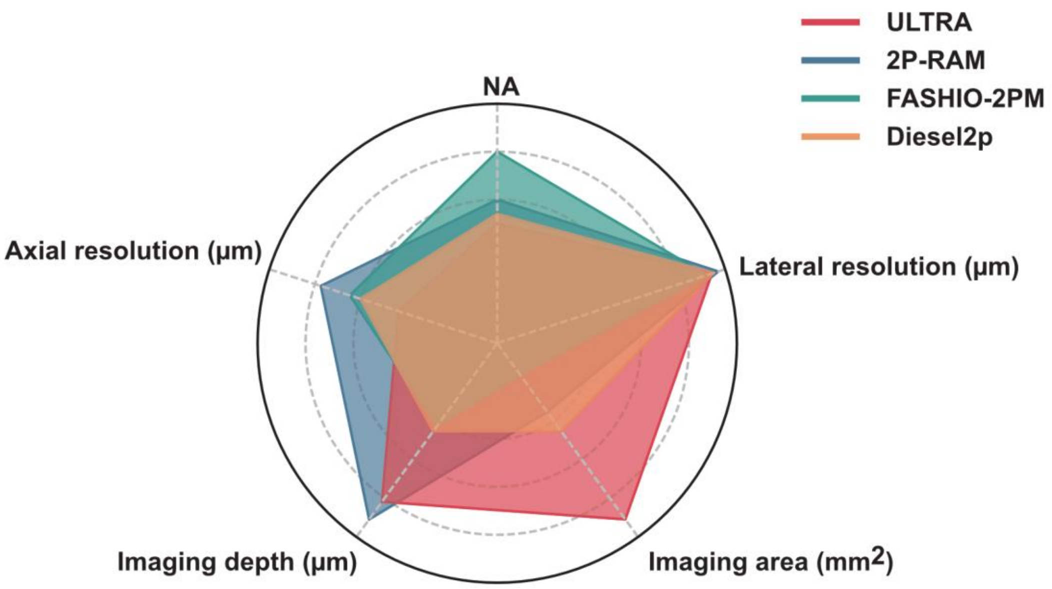

Supplementary Fig. 10 Comparison of ULTRA with several other recently developed two-photon mesoscopes. Performance comparison of ULTRA with state-of-the-art multi-photon systems. The radar chart summarizes five key optical performance metrics (all the values are ranged from the center outwards): Numerical Aperture (NA, range 0 - 1), Lateral resolution (μm, range 20 - 0), Imaging area (mm^2, range 0 - 55), Imaging depth (μm, range 0 - 1100), and Axial resolution (μm, range 20 - 0). ULTRA (red) demonstrates a significant advantage in imaging area while maintaining high NA and spatial resolution, effectively achieving a higher space-bandwidth product (SBP) compared to 2P-RAM (blue), FASHIO-2PM (green), and Diesel2p (orange).

Supplementary Video 1:
Ultra imaging on vasculature from 4 regions up to 870 μm deep in Fig. 3d. Frame rate: 6.02 Hz

Supplementary Video 2:
Ultra calcium imaging in Fig. 4a

Supplementary Video 3:
Ultra calcium imaging in Fig. 4b

Supplementary Video 4:
Ultra calcium imaging in Fig. 4d (7.07 Hz)

Supplementary Video 5:
Ultra calcium imaging in Fig. 4d (16.67 Hz)

Supplementary Video 6:
Ultra calcium imaging in Fig. 4g
